# Development and psychometric properties evaluation of caregiver burden questionnaire in family caregivers of hemodialysis patients

**DOI:** 10.1186/s12912-022-01025-7

**Published:** 2022-09-05

**Authors:** Sima Sadat Hejazi, Meimanat Hosseini, Abbas Ebadi, Hamid Alavi Majd

**Affiliations:** 1 Department of Community Health Nursing, School of Nursing and Midwifery, Shahid Beheshti University of Medical Sciences, Tehran, Iran; 2 Department of Nursing, Bojnurd Faculty of Nursing, North Khorasan University of Medical Sciences, Bojnurd, Iran; 3 Department of Community Health Nursing, School of Nursing and Midwifery, Shahid Beheshti University of Medical Sciences, Vali Asr Ave, Niayesh Cross Road, Niayesh Complex , Tehran, Iran; 4 Behavioral Sciences Research Center, Life Style Institute, Faculty of Nursing, Baqiyatallah University of Medical Sciences, Tehran, Iran; 5 Department of Biostatistics, School of Allied Medical Sciences, Shahid Beheshti University of Medical Sciences, Tehran, Iran

**Keywords:** Caregivers, Caregiver burden, Hemodialysis, Questionnaire

## Abstract

**Backgorund:**

This study aimed to design and psychometrically evaluate the caregiver burden questionnaire for family caregivers of hemodialysis patients.

**Methods:**

This study was conducted using an exploratory sequential mixed method on family caregivers of hemodialysis patients in Iran. In the first phase, the generation of the items was done based on results of directed qualitative content analysis according to the Structural model of the caregiver burden and review of the literature. After developing the item pool, face and content validity, item analysis, structural, convergent and discriminant validity, internal consistency, reliability, interpretability, and feasibility were evaluated.

**Results:**

The primary tool entered the psychometric evaluation phase with 64 items. After performing face and content validity and item analysis, the number of items was reduced to 28. Exploratory factor analysis was performed with 28 items and 300 caregivers, and finally, four subscales with 21 items were developed. The results of confirmatory factor analysis indicated a good fit of the model. Cronbach's alpha and the Intraclass Correlation Coefficient of all subscales were higher than 0.7 and 0.9, respectively. The standard error of measurement was 1.39. All subscales had acceptable values in convergent validity criteria and the HTMT index less than the threshold value. The total score of the questionnaire had no ceiling and floor effect; the percentage of unanswered items was within the acceptable range.

**Conclusion:**

The results show that the caregiver burden questionnaire for caregivers of patients undergoing hemodialysis has good psychometric properties and can measure the caregiver burden in these caregivers.

**Supplementary Information:**

The online version contains supplementary material available at 10.1186/s12912-022-01025-7.

## Background

By the end of 2017, 697.5 million patients with chronic kidney disease were reported worldwide [1] and the number of people in the world who have been treated with kidney replacement therapy has reached more than 2.5 million patients. This number is expected to increase to 5.4 million by 2030 [1, 2]. Also, until 2015, more than 27,000 patients were treated in 500 hemodialysis centers in Iran [3].

Patients undergoing hemodialysis encounter specific problems, such as the need for regular hemodialysis sessions with relatively long hours and dependence on this treatment, adherence to a complex dietary regimen and restrictions and consumption of multiple medications [4, 5]. Also, complications related to vascular access and necessitating frequent visits, diagnostic tests, and hospitalization because of the nature of chronic kidney disease, are other specific issues in these patients along with sexual problems [3, 6]. In this situation, except for the patient, the person who is most affected by the disease and treatment process is the family caregiver [7].

Caregiver burden is defined as the “degree of physical, emotional, financial, and social suffering that a family caregiver perceives while caring for the patient [8]”. A family caregiver is an individual who provides ongoing and unpaid care and assistance to a family member or friend who is physically, cognitively, or mentally disabled [9]. There is a lot of emphasis on investigating the caregiver burden among family caregivers to improve their quality of life and general well-being [10]. Understanding caregivers’ conditions can help healthcare professionals provide treatment process support and psychological care to hemodialysis patients [3, 11]. The early identification of caregivers who perceive burden is one of the important elements in developing clinical programs to meet the medical and supportive care needs of caregivers and increase their level of knowledge and awareness. In addition, an accurate measurement of caregiver burden is essential for doing clinical research in this area [12].

There are limited studies providing information about the lives of family caregivers of hemodialysis patients and addressing caregiver burden among them [3, 11]. Most of the studies on caregiver burden have addressed caregivers of patients with neurological disorders [13] on the other hand, most studies on the measurement of caregiver burden among caregivers of patients undergoing hemodialysis used general tools for measuring caregiver burden, such as the Zarit Burden Interview (ZBI), Caregiver Burden Scale (CBS(, and Oberst Caregiving Scale [11, 14]. These tools are primarily designed to measure caregiver burden in neurological and cognitive disorders patients. In addition, they measure general aspects along with other existing tools for assessing caregiver burden. Nevertheless, it seems that these tools do not address all aspects of caregiver burden in caregivers of hemodialysis patients.

On the other hand, several studies have developed specific tools for measuring caregiving burden in different groups in recent years. For example, Arlandis et al. (2017) designed and assessed the psychometric properties of a caregiving burden scale in close relatives of patients with an Overactive Bladder (OAB). Their scale included dimensions of irritability, sexual function, travel, and anxiety [15]. Mortenson et al. (2017) also developed a tool to measure caregiving burden in family caregivers of powered wheelchair users, including two general dimensions of device-specific and overall caregiver burden [16]. To the best of our knowledge, there are no specific tools for measuring caregiver burden in caregivers of hemodialysis patients. We only found one study that did a psychometric analysis of a tool in caregivers of hemodialysis patients. In the study mentioned above, Cil Akinci et al. (2012) psychometrically assessed a caregiver burden scale in caregivers of patients undergoing hemodialysis [13]. The scale had 22 items, and it was initially developed to measure the caregiving burden in caregivers of stroke patients. It included general strain, isolation, disappointment, emotional involvement, and environment subscales [17]. The special needs and problems of the patient undergoing hemodialysis and, consequently, the different issues that the caregivers face necessitate the need to develop a specific tool to assess the caregiver burden in the caregivers of these patients. Having a specific tool to assess the caregiving burden in family caregivers of hemodialysis patients can help measure and identify the actual caregiving burden and make supportive decisions to reduce this burden. Identifying the actual dimensions of the caregiving burden leads to more targeted planning for support service providers. Consequently, the reduction of caregiving burden in family caregivers can have positive consequences, such as improving their quality of life and general health, which, in turn, has positive direct and indirect effects on the hemodialysis patients' conditions. The aim of this study was the development and psychometric properties evaluation of a caregiver burden questionnaire in family caregivers of hemodialysis patients.

## Methods

This study was conducted using an exploratory sequential mixed method design from May 2020 to May 2021. The study consisted of two phases: 1) qualitative content analysis and literature review for item generation and 2) a methodological study for psychometric evaluation of the questionnaire.

### Qualitative content analysis and literature review for item generation

The generation of the initial items was done based on extracted codes, subcategories, generic categories, and main categories through performing directed qualitative content analysis according to the Structural model of the caregiver burden model [18, 19] and review of the literature. The literature review was carried out based on the the Preferred Reporting Items for Systematic Reviews and Meta-Analyses (PRISMA) [20] to identify current tools for measuring informal or family caregiver burden. The databases that were Web of Knowledge, Scopus, MEDLINE, CINAHL, PsycINFO and ProQuest also the reference lists of relevant included studies, thesis and conference proceedings had been searched. All studies in which a new instrument had been developed or the main goal of the authors was the psychometric evaluation of a tool via classic test theory was included [21].

### Methodological study

#### Face validity

First, quantitative then quailitative face validity was examined. For this purpose, the scale was provided to 10 family caregivers of patients undergoing hemodialysis, and the item impact score (IIS) was calculated. After that, to evaluate the qualitative face validity, face-to-face interviews were conducted. The caregivers with different levels of literacy and socioeconomic were selected through purposive sampling method. For this purpose, sampling was done from different hospitals in different regions of the Tehran city and caregivers with different levels of literacy (from elementary to academic), forms of relations with the patient (wife, child or others) and years of providing care were included in this phase of the study.The inclusion criteria were the ability to speak Farsi, being responsible for the main care of the patient, not being paid for providing care, and caring for patients over 18 years of age.

For calculation of IIS, each caregiver was asked to select one of the choices, including the most appropriate (score 5), to some extent appropriate (score 4), fairly appropriate (score 3), slightly appropriate (score 2), and not appropriate (score 1) for each item. The IIS was calculated by multiplying the number of people who rated the item 4 or 5 and the mean score of the item. If the IIS of an item was equal to or greater than 1.5, it was appropriate [22]. To evaluate the qualitative face validity, the caregivers were asked to evaluate each item's level of difficulty, relevancy, and ambiguity.

#### Content validity

First, qualitative content validity was assessed. To do so, the scale was provided to 13 experts, including four researchers in the field of hemodialysis, four researchers familiar with the caregiving burden concept, two experts in psychometric evaluation, a psychiatrist, a researcher in the field of health education, and a linguist. They were asked to comment on the grammar, wording, item allocation, and scaling. For assessing the quantitative content validity, the following steps were done. To calculate the Content Validity Ratio (CVR‌), 14 experts, including professionals with experience in hemodialysis, experts familiar with the concept of caregiving burden and psychometric evaluation, a psychiatrist, and a nurse with 20 years of work experience in the dialysis unit were asked to rate each item as essential, useful but not essential, and not necessary. Since the number of experts was 14, items with a CVR < 0.51 had to be omitted [18]. Then, to determine Individual Item Content Validity Index (I-CVI), the scale was provided to 10 experts to score each item from 1 to 4 (1 = not relevant, 2 = somewhat relevant, 3 = relevant, and 4 = very relevant). After calculating I-CVI, the modified kappa statistics were determined. Using the criterion proposed by Fleiss (1981), according to which kappa values above 0.75 are considered excellent [22], Polit et al. (2007) showed that any I-CVI value higher than 0.78 would be equal to a modified kappa higher than 0.75. Thus, it can be considered evidence suggesting that the question is sufficiently relevant [23]. Then, the Scale-Content Validity Index (S-CVI) was calculated using the average CVI. The criterion for confirming S-CVI was 0.9 [22].

#### Item analysis

The questionnaire was given to 30 family caregivers of patients undergoing hemodialysis for item analysis. The caregivers were selected through convenience sampling method from academic medical centers of Tehran, Iran. The Cronbach's Alpha coefficient of the questionnaire, inter-item correlation, Cronbach's Alpha if item deleted, and item-total correlation were calculated. Items with a correlation greater than 0.7 were merged with another item, and items with an item-total correlation of less than 0.2 were removed. In addition, if Cronbach's alpha increased by deleting an item, that item would also be removed.

#### Construct validity

The Exploratory Factor Analysis (EFA) and Confirmatory Factor Analysis (CFA) were used to assess construct validity with two separated samples (300 individuals for EFA and 320 individuals for CFA through comvenience sampling method). The samples have completed the 28-item questionnaire. In decision-making regarding the required sample size in factor analysis, rules have been formulated that include the absolute minimum sample size (100 participants), participants per variable ratio (10 to one) and variable to expected factors ratio (at least 3 to 1). Other studies consider the factors affecting the sample size like common variance estimation and factor loadings and overdetermination [24]. According to the COnsensus-based Standards for the selection of health status Measurement INstruments (COSMIN) checklist, a sample size of seven times the number of items and more than 100 is a very good sample size for factor analysis [25, 26]. In this research, according to a combination of existing criteria and taking into account that the input questionnaire for EFA and CFA had 28 and 21 items, respectively, factor analysis was performed with 300 and 320 questionnaires completed by caregivers.

In this phase, both paper and pencil and online format of the questionnaire were used for data collection. The online questionnaire was made via an Iranian platform; the Uniform Resource Locator (URL) of the online questionnaire format was sent to family caregivers by social networks such as Whatsapp and email all over the country. The number and emails of the caregivers were obtained by contacting the head nurses of hemodialysis wards. Also, researchers referred to academic medical centers of Tehran, Iran, to collect the data via paper and pencil format. The inclusion criteria for caregivers were: not having a mental disorder such as depression and anxiety, caregiving to a patient over the age of 18, not being paid for caregiving, and their patients must not be residents in nursing homes or hospices. Before performing factor analysis, the normal distribution of data (univariate normality by skewness between -3 and + 3 and Kurtosis between -7 and + 7; multivariate normality by examining Mardia's coefficient less than 20) and the presence of outliers in the data (by scatterplots and boxplots in the EFA; by Mahalanobis distance in CFA) were investigated. Scree plots and eigenvalues were used to decide how many factors should be maintained (only the significance of factors with eigenvalues equal to or greater than one was investigated, and those with eigenvalues less than one were ignored). Bartlett's test should be significant, and Kaiser–Meyer–Olkin (KMO) > 0.8 is appropriate [27]. The critical value for maintaining the item in the factor was considered to be 0.3, which was obtained by the following formula:

$$\mathrm{cv}=5.152\surd\left(\mathrm n-2\right)$$

In this formula, *n* equals the sample size [28]. Items with a communality less than 0.2 were also removed [29]. In addition, having at least three items per factor was observed.

CFA was performed with the maximum likelihood method, and goodness-of-fit indices of Adjusted Goodness-of-Fit Index (AGFI), Comparative Fit Index (CFI), Incremental Fit Index (IFI), Parsimonius Comparative Fit Index (PCFI), Parsimonius Normed Fit Index (PNFI), Root Mean Square Error of Approximation (RMSEA), and minimum discrepancy function divided by degree of freedom (CMIN/DF) were evaluated. All statistical analyses were performed using SPSS_24_ and AMOS_24_.

#### Convergent and discriminant validity

The convergent validity and discriminant validity were assessed with CFA samples. The following criteria were used to assess convergent and discriminant validity based on Fornell-Larcker criteria:

##### ***Convergence validity***

Standard factor loads greater than 0.5, Composite Reliability (CR) greater than Average Variance Extracted (AVE), and AVE greater than 0.5.

##### ***Discriminant validity***

Maximum Shared Variance (MSV) less than AVE [29].

In addition to the mentioned criteria, the Heterotrait-Monotrait (HTMT) ratio method was used to evaluate the discriminant validity. In this study, the criterion of 0.90 was considered [30]. To calculate the Fornell-Larcker criterion and the HTMT ratio, a Plugin that could be installed on AMOS and was designed by Professor James Gaskin [31] was used.

#### Internal consistency and reliability

For evaluation of internal consistency, Cronbach's alpha and Average inter-item Correlation (AIC) were calculated (based on the data of EFA (sample size: 300)). Alpha coefficient > 0.7 [32] and AIC between 0.2 and 0.4 [28] were considered acceptable. For reliability evaluation, the Intraclass Correlation Coefficient (ICC) of the questionnaire was assessed by a two-way mixed model with absolute agreement. At this stage, 50 caregivers (the sample size based on Terwee et al., recommendation) [33] who were selected through convenience sampling method from academic medical centers of Tehran, Iran, completed the questionnaire twice, two weeks apart The questionnaire was completed both online and through self-reporting each time. Before completing the questionnaire in the retest phase, an oral interview was conducted with the caregivers to ensure that the caregivers and their patients’ conditions were the same as those in the test phase and that no unexpected event had occurred that caused a change in the caregiving burden. For assessment of Composite Reliability (CR) (based on the data of CFA), the criteria of CR > 0.7 or H coefficient > 0.7 were considered [29].

#### Standard error of measurement, minimal detectable change, minimal important change, and interpretability

The Standard Error of the Measurement (SEM) was calculated based on the data of reliability phase with the following formula in which SD means the standard deviation of the sum of the test and retest values (SD POOLED) and ICC is the stability reliability [23]:

$$SEM=SD\times \sqrt{\left(1-ICC\right)}$$

In addition, the Minimal Detectable Change (MDC) was obtained according to the following formula [22]:

$${MDC}_{95}=SEM\times\sqrt2\times\;1.96$$

The Minimal Important Change (MIC) was calculated by the following formula in which the SD of the changes between test–retest was multiplied by an average effect size of 0.5 [34]:

$$\mathrm{MIC}=0.5\times\mathrm{SD}\;\mathrm{of}\;\mathrm{the}\;\Delta\;\mathrm{Score}$$

The Limit of Agreement (LOA) was calculated based on the following formula, in which *d* is the average of changes in the two evaluation stages [23]:

$$\mathrm{LOA}=\mathrm d\pm1.96\times\mathrm{SD}\;\mathrm{difference}$$

For the interpretability, the ceiling and floor effect was evaluated [35] for the first 400 samples out of 620 completed questionnaires in the the construct validity assessment phase (EFA and CFA). The ceiling and floor effect occurs when more than 15% of respondents achieve the lowest or highest possible score. The presence of ceiling and floor effects indicates the low content validity of the tool [33].

#### Feasibility

To evaluate the feasibility of the tool, the response time and the number of missed items were assessed [36]. Missed items up to 15% are expected in educational and psychological studies [37].

## Results

### Item generation

According to the qualitative study results and the literature review, an item pool containing 479 primary items was developed. The research team examined this item pool in seven phases. Similar or overlapping items were removed or merged, and eventually, the primary tool consisting of 64 items was developed. An additional file shows this in more detail [see Additional file 1].

### Face validity

In the quantitative face validity phase, nine items had IIS less than 1.5 that were removed. Then, the necessary modifications were made following the family caregivers’ opinions obtained in the qualitative face validity phase.

### Content validity

Due to overlapping, twelve items were removed or merged with other items in the qualitative content validity phase. Finally, the number of items was reduced to 43 items. According to experts’ opinions and based on Lawshe's criterion, ten items with CVR < 0.51 were omitted in the quantitative phase. Moreover, after calculating the modified kappa in the phase of I-CVI calculation, only one item was removed due to kappa < 0.75. Besides, S-CVI calculated using the average CVI was 0.96.

### Item analysis

The Cronbach's alpha and standardized Cronbach's alpha were 0.871 and 0.871, respectively. No items had a correlation coefficient above 0.7. Four questions had item-total correlation less than 0.2, which were omitted.

### Construct validity

The participants’ demographic characteristics in the EFA phase are shown in Table 1.

**Table 1:** Demographic characteristics of participants (*N* = 300)

**Demographic variables**		**Number (%) **
**Caregiver Gender**	Female	190(63.5)
	Male	109 (36.5)
**Caregiver marital status**	Married	204 (68)
	Single	87 (29)
	Others	7 (23)
**Relationship to the patient**	Patient’s child	166 (56.1)
	Spouse	56 (18.9)
	Others	35 (11.7)
	Patient’s mother	18 (6.1)
	Patient’s father	7 (2.4)
	Patient’s sister	7 (2.4)
	Patient’s brother	7 (2.4)
**Caregiver age**		37.95 (12.82) ^*^
**Patient age**		54.69 (16.45) ^*^
**Duration of treatment with hemodialysis**		4.11 (4.02) ^*^

* Mean (SD)

**Fig. 1:**
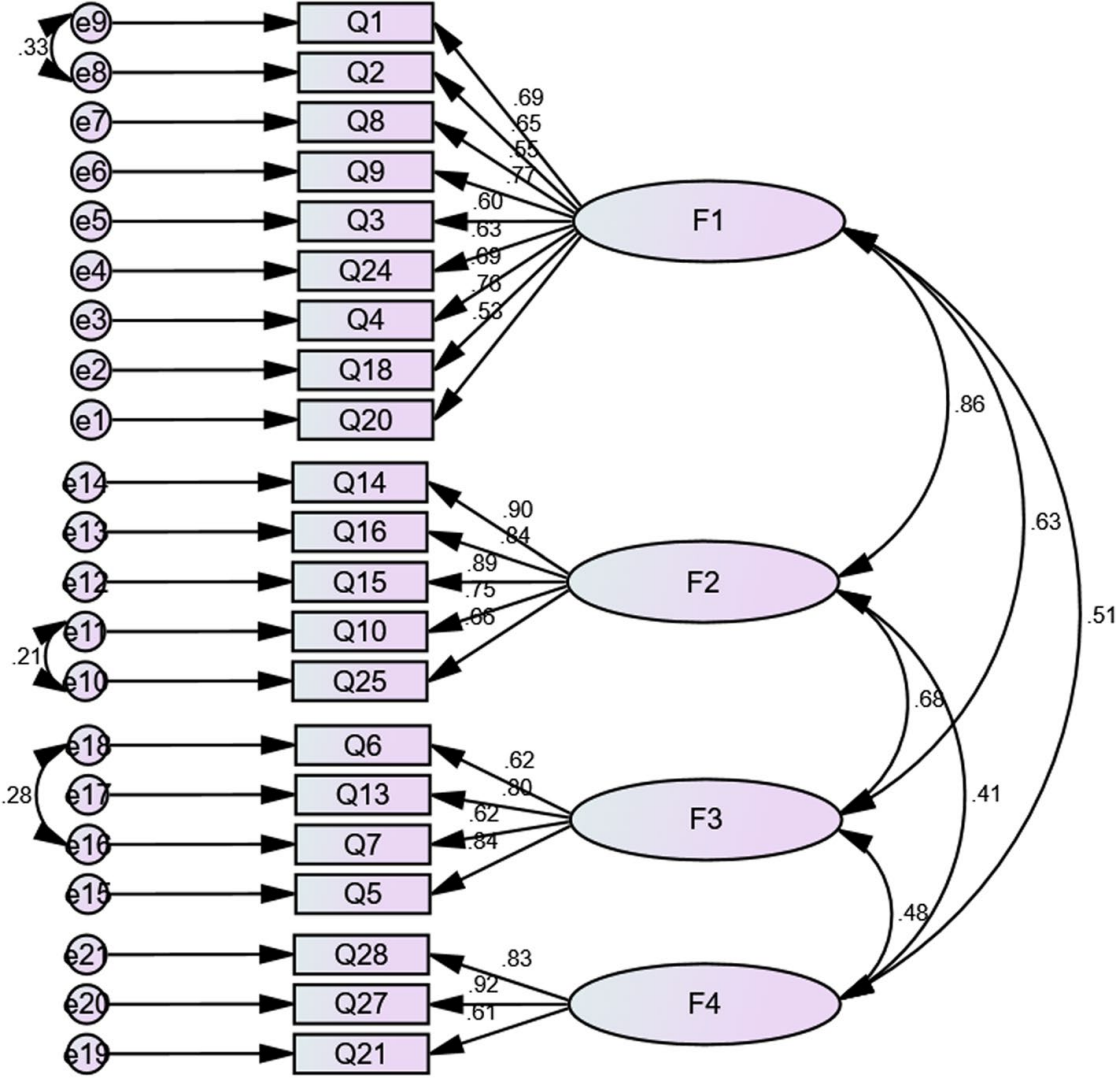
The final structure model of the caregiver burden questionnaire

Twenty-eight items entered EFA. An additional file shows this in more detail [see Additional file 2]. The final questionnaire after EFA consisted of 21 items and four factors, labeled as physical and psychological burden (nine items), social-time burden (five items), mental burden (four items), and supportive-financial burden (three items). The KMO was equal to 0.919, and Bartlett’s test was 3135.899 (*P* < 0.001) (Table 2). In the CFA, after adjusting the model (three pairs of measurement errors between items one and two, ten and twenty-five, and six and seven), goodness-of-fit indices were calculated, shown in Table 3. In addition, the modified model of the concept of caregiver burden resulting from the CFA is shown in Fig. 1.

**Table 2:** Four extracted factors of the caregiver burden questionnaire and their factor loadings (*N* = 300)

**Subscales**	**Items**	**Factor loading**	**Communality**	**Eigenvalue**	**Variance explained**
**Physical and psychological burden**	1- My physical health is endangered due to taking care of my patient	0.837	0.611	3.0072	14.32
	2- Accompanying my patient in the hemodialysis center makes me tired	0.707	0.515		
	8- I feel I can no longer continue to take care of my patient	0.695	0.386		
	9- I do not sleep well because of taking care of my patient	0.604	0.579		
	3- Because of my patient, my diet has been disrupted	0.481	0.461		
	24- The burden of caring for my patient has reduced my sexuality	.0475	0.455		
	4- Taking care of my patient has made me feel depressed	.0455	.0481		
	18- After starting hemodialysis, I am responsible for my patient and my own life	0.387	0.474		
	20- The non-cooperation of other family members in caring has imposed a significant burden on me	0.381	0.376		
**Social-time burden**	14.- My recreation has decreased due to taking care of my patient	0.937	0.829	2.8699	13.666
	16- I can travel less than before	0.908	0.720		
	15- My relationships with others have diminished because of caring for my patient	0.80	0.725		
	10- My patient's hemodialysis schedule determines my life plan	0.622	0.535		
	25- One of my difficulties is monitoring my patient’s medication and nutrition	0.375	0.405		
**Mental burden**	6- I am worried that I have failed to take care of my patient	0.810	0.552	1.9350	9.214
	13- I am afraid of my patient’s future	0.692	0.608		
	7- I feel that I cannot do anything about my patient's problems	0.635	0.477		
	5- I am constantly thinking about my patient	0.630	.0546		
**Supportive-financial burden**	28- I cannot afford my patient’s care	0.886	0.678	1.8516	8.817
	27- My patient's lack of full health insurance coverage has imposed a financial burden on me	0.871	0.748		
	21- The weakness of the support system (government and charity) for the patient and caregiver has burdened me	0.555	0.453

**Table 3:** The fit model indices of confirmatory factor analysis of caregiver burden questionnaire

**Chi-Square, df, *P*-value**	**CMIN/DF**	**RMSEA**	**PNFI**	**PCFI**	**IFI**	**CFI**	**AGFI**
489.259, 180, *p* < 0.001	2.718	0.074	0.752	0.787	0.919	0.918	0.839

### Convergent and discriminant validity

Although subscale one gained an AVE of less than 0.5, it achieved acceptable values in other convergent validity criteria. Other subscales also had acceptable values in convergent validity criteria. Regarding discriminant validity, according to the Fornell-Larcker criterion, subscales three and four achieved the necessary criterion, i.e., MSV < AVE. However, subscales one and two did not have MSV < AVE. Based on the results, the HTMT index was less than the threshold value, i.e., 0.9, for all subscales; therefore, the presence of acceptable discriminant validity was ensured (Tables 4 and 5).

**Table 4:** Convergent, discriminant validity and composite reliability of caregiver burden questionnaire

**Subscales**	**CR**	**AVE**	**MSV**
Physical and psychological burden	0.872	0.433	0.737
Social-time burden	0.905	0.660	0.737
Mental burden	0.814	0.527	0.461
Supportive-financial burden	0.836	0.636	0.263

**Table 5:** HTMT of caregiver burden questionnaire

**Subscales**	**Subscale 1**	**Subscale 2**	**Subscale 3**	**Subscale 4**
**Subscale 1**				
**Subscale 2**	0.84			
**Subscale 3**	0.63	0.69		
**Subscale 4**	0.55	0.49	0.51	

### Internal consistency and reliability

Cronbach's alpha of all the subscales of the caregiving burden questionnaire was higher than 0.7. The overall ICC of the tool was 0.98 (CI 95%: 0.97,0.99). Moreover, all subscales had acceptable CR. Details of the results are shown in Table 6.

**Table 6:** Internal consistency and reliability of caregiver burden questionnaire

**Subscales**	**Alpha**	**AIC**	**ICC**	**ICC 95%**	**MaxR(H)**
Physical and psychological burden	0.87	0.438	0.98	0.97, 0.99	0.883
Social-time burden	0.87	0.583	0.96	0.93, 0.97	0.936
Mental burden	0.77	0.462	0.96	0.93, 0.97	0.843
Supportive-financial burden	0.80	0.573	0.97	0.96, 0.98	0.983

### Standard error of measurement, minimal detectable change, minimal important change, and interpretability

Based on the results, the SEM was 1.39, the MDC was 3.85, and the MIC was 1.109. The LOA was 4.22–4.46. In a sample of 400 questionnaires completed by family caregivers of hemodialysis patients, the frequency of the participants with the maximum and minimum total score was zero.

### Feasibility

The response time was 7:00 min for the online questionnaire and five minutes and 50 s for the paper and pencil format questionnaire. The percentage of unanswered items for the whole questionnaire was 2.08%, and the ratio of answered items was 97.92%.

### Scoring

The caregiving burden questionnaire was a self-reporting one, which was scored using a five-point Likert scale (1 = very low, 2 = low, 3 = average, 4 = high, and 5 = very high). To better understand the scoring and comparability of scores, all scores were converted to a scale of 0–​100.

This conversion was done using the linear conversion formula. The higher the score, the greater the caregiving burden:

$$\mathrm{Factor}\;\mathrm{score}=\frac{\mathrm{obtained}\;\mathrm{score}\;\mathrm{from}\;\mathrm{each}\;\mathrm{factor}-\mathrm{minimum}\;\mathrm{score}}{\mathrm{maximum}\;\mathrm{score}-\mathrm{minimum}\;\mathrm{score}}\times100$$

## Discussion

This study aimed to develop and evaluate the psychometric properties of a caregiving burden questionnaire in family caregivers of patients undergoing hemodialysis. The results showed that this questionnaire had good validity, reliability, and internal consistency. This questionnaire included 21 items and four subscales: physical and psychological burden, social-time burden, mental burden, and supportive-financial burden. In this study, reliability was measured by calculating different indices. In addition, the SEM, MDC, and LOA recommended by the COSMIN checklist were calculated.

Moreover, the MIC and the ceiling and floor effects were evaluated, which have not been studied in many caregiver burden tools. The first subscale, physical and psychological burden, included nine items. Many caregiving burden scales have this subscale. In the Caregiver Burden Inventory (CBI) developed by Novak and Guest (1989), physical and psychological burdens are two separate subscales. In the physical subscale of CBI, sleep disorders, fatigue, and physical problems have been addressed [38].

Similarly, in the present questionnaire, items one, two, and four of the first subscale addressed these issues. In the subscale of psychological (emotional) burden, in CBI, the focus is on the problems that have occurred due to the behaviors of the patient with cognitive impairment, such as feeling ashamed of the patient’s behaviors, feeling angry about the reactions toward the patient, and feeling uncomfortable [38], which are different from psychological problems addressed in our first subscale. The initial version of ZBI (1986), an old and well-known scale measuring caregiving burden, has 29 items and no subscale. In the scale mentioned earlier, there is an item related to feeling depressed in the relationship with the spouse [8], which is somewhat similar to the item of feeling depressed due to caregiving in the present questionnaire. Similar to our questionnaire, the effect of caregiving on the caregivers’ health is addressed in ZBI (but only with one item).

Regarding physical, psychological issues, only items about feeling fatigue and health-related suffering are mentioned in the Caregiver Burden Scale (CBS) designed by Elmstahl et al. (1996) [17]. On the contrary, in the physical and psychological subscale of our questionnaire, the impacts of care on the physical and mental health aspects of the caregiver were specifically and precisely addressed. Some of these health-related consequences were fatigue, nutritional problems, depression, sleep disorders, and sexual problems, especially the latter one, which is not considered in many of the tools measuring the caregiving burden.

The social-time burden subscale included five items addressing issues, such as restricted recreation, travel, social activities, and communication with others, which are also considered in other tools in this field [17, 38]. However, it seems that items, such as the determining role of the patient's hemodialysis schedule in the caregiver's life plan (item 4) and requiring a long time for monitoring the patient's diet and medication (item 5), are specific to the caregivers of hemodialysis patients and are not presented in other available tools. The subscale of mental burden referred to anxiety about the future, mental involvement with care, and a sense of failure in care. In ZBI, the caregiver’s fear of the future is also questioned [8]. These issues have received less attention in CBI and CBS [17, 38]. The patient undergoing hemodialysis is struggling with extensive and complex physical and psychological problems. The chronic nature of the disease, the patient's permanent dependence on a kidney replacement therapy, therapeutic complexities, and complications in the patient can impose a mental burden on the caregiver, leading to the consideration of this item in our questionnaire. In the subscale of supportive-financial burden, difficulty in financing costs, financial burden imposed by low insurance coverage, and lack of support were questioned. In ZBI, this issue is examined in the form of one item [8]. On the contrary, CBI and CBS have not considered the financial burden [17, 38].

One of the strengths of this research was extensive sampling from most cities of Iran due to the use of both paper and online questionnaires. Another strength of this study was the evaluation of other psychometric properties of the questionnaire and validity and reliability. One of the limitations of this study was that the majority of family caregivers were female, which may reduce the generalizability of the study findings. Also, the convenience sampling method can reduce the generalizability of the findings. Another limitation of this study was that we did not assess the responsiveness of the questionnaire due to time limitations.

## Conclusion

The caregiver burden in family caregivers is inevitable. However, its early identification through appropriate tools, planning, implementing appropriate interventions, and family caregiver support can be helpful. This caregiver burden questionnaire developed for family caregivers of hemodialysis patients can measure caregiver burden in this group of caregivers by addressing the general and specific aspects of this burden using a small number of questions (21 questions). Future studies are proposed to investigate this questionnaire in family caregivers of patients undergoing hemodialysis with different cultures.

## Supplementary Information

**Additional file 1**. The primary questionnaire at the beginning of psychometric assessment step.

**Additional file 2.** The 28-items questionnaire at the end of item analysis step.

## Data Availability

The datasets used and analyzed during the current study are available from the corresponding author on reasonable request.

## Abbreviations

CBS: Caregiver Burden Scale
IIS: Item Impact Score
CVR‌: Content Validity Ratio
I-CVI: Item Content Validity Index
S-CVI: Scale-Content Validity Index
EFA: Exploratory Factor Analysis
CFA: Confirmatory Factor Analysis
URL: Uniform Resource Locator
KMO: Kaiser–Meyer–Olkin
AGFI: Adjusted Goodness-of-fit Index
PCFI: Parsimonius Comparative Fit Index
PNFI: Parsimonius Normed Fit Index
CFI: Comparative fit index
IFI: Incremental fit index
RMSEA: Root mean square error of approximation
CMIN/DF: Minimum discrepancy function divided by degree of freedom
CR: Composite Reliability
AVE: Average Variance Extracted
MSV: Maximum Shared Variance
HTMT: Heterotrait-Monotrait
AIC: Average inter-item Correlation
ICC: Intraclass Correlation Coefficient
SEM: Standard Error of the Measurement
MIC: Minimal Important Change
LOA: Limit of Agreement
COSMIN: COnsensus-based Standards for the selection of health status Measurement INstruments
CBI: Caregiver Burden Inventory
ZBI: Zarit Burden Interview
