# Supplementary material for: Development and psychometric properties evaluation of caregiver burden questionnaire in family caregivers of hemodialysis patients
Source: BMC Nurs. 2022 Sep 5;21:246. doi: 10.1186/s12912-022-01025-7 (PMC9446771; doi:10.1186/s12912-022-01025-7)
Supplement: Supplementary file 1 — Additional file 1. The primary questionnaire at the beginning of psychometric assessment step. [file 12912_2022_1025_MOESM1_ESM.docx]

Additional file 1: The primary questionnaire at the beginning of psychometric assessment step

| 1 | Paying my patient’s care expenses has put pressure on me. |
| --- | --- |
| 2 | My patient’s medical expenses are not fully covered by insurance. |
| 3 | The decline in my patient’s income has put pressure on me. |
| 4 | My physical health is at risk due to giving care to my patient. |
| 5 | Accompanying my patient at the hemodialysis center exhausts me. |
| 6 | My and my family members’ diet has been restricted. |
| 7 | I cannot take care of my health. |
| 8 | I worry about fistula failure or a pericardial infection in my patient. |
| 9 | The slightest change in my patient’s condition worries me. |
| 10 | I feel the symptoms of depression in myself. |
| 11 | Supplying medicine to my patient is one of my concerns. |
| 12 | I cannot leave the care of my patient to another person due to worries about putting pressure on others. |
| 13 | I am worried that my patient will be delayed at the hemodialysis center. |
| 14 | Providing a vehicle to get to the hemodialysis center is my concern. |
| 15 | One of my concerns is my patient’s kidney transplant. |
| 16 | I am in low spirits seeing my patient suffer. |
| 17 | I am constantly preoccupied with my patient. |
| 18 | I feel I have failed to take good care of my patient. |
| 19 | I feel guilty about my behavior toward my patient. |
| 20 | I feel like I can do nothing about my patient’s problems. |
| 21 | I feel I can no longer take care of my patient. |
| 22 | I do not get quality sleep due to taking care of my patient. |
| 23 | The strain of taking care of my patient has reduced my sexual desires. |
| 24 | My patient cooperates with me in adhering to the diet. |
| 25 | My patient has high expectations of me. |
| 26 | My patient blames me for the limitations caused by his/her illness. |
| 27 | Taking care of my patient is a simple task. |
| 28 | My patient’s hemodialysis schedule determines my life plans. |
| 29 | I feel that I have to constantly control everything. |
| 30 | My life has become restricted due to taking care of my patient. |
| 31 | I have to pretend that my patient’s dialysis has not caused me any problems. |
| 32 | I have to endure the current unfavorable situation. |
| 33 | I have problems performing religious acts due to my involvement in care. |
| 34 | I am stressed that there is no one or place to answer my questions about my patient. |
| 35 | I am worried about the future of my life. |
| 36 | I am afraid of what the future holds for my patient. |
| 37 | I am in low spirits due to my prolonged presence in the hospital. |
| 38 | I am worried that my patient’s dialysis will not be of the required quality. |
| 39 | My recreation has been limited due to giving care to my patient. |
| 40 | My social life has been limited due to taking care of my patient. |
| 41 | My family life has been affected by my patient’s problems. |
| 42 | My married life has been affected by the care of my patient. |
| 43 | I cannot take good care of my spouse and children due to caring for my patient. |
| 44 | I can travel less than before. |
| 45 | I have lost my job opportunities or been forced to retire. |
| 46 | I cannot do my job efficiently due to taking care of my patient. |
| 47 | After my patient’s hemodialysis, my patient’s and my life’s responsibilities have fallen on me. |
| 48 | I am annoyed by others’ sympathetic statements. |
| 49 | I feel that others do not understand my problems caring for my patient. |
| 50 | I am under pressure because other family members do not cooperate in the care. |
| 51 | I am strained because others perceive that my patient’s problem is inherited. |
| 52 | Others’ perceptions of my patient being infected restrict me. |
| 53 | I have been abused in taking care of my patient. |
| 54 | I have not been appreciated for taking care of my patient. |
| 55 | My patient’s problems have caused a conflict between other caregivers and me. |
| 56 | My relatives have high expectations of me. |
| 57 | The lack of a support system for the patient and caregiver puts pressure on me. |
| 58 | The hospital staff does not sufficiently understand and empathize with me about my patient. |
| 59 | Ward staff’s inadequate involvement has resulted in my distrust of them. |
| 60 | All my time is devoted to taking care of my patient. |
| 61 | My patient is able to take care of himself/herself on his/her own. |
| 62 | One of my problems is monitoring my patient’s medicine consumption and nutrition. |
| 63 | I have to accompany my patient to the dialysis center due to his/her condition. |
| 64 | One of my problems is the side effects of kidney failure in my patient. |
