# Supplementary material for: Development and psychometric properties evaluation of caregiver burden questionnaire in family caregivers of hemodialysis patients
Source: BMC Nurs. 2022 Sep 5;21:246. doi: 10.1186/s12912-022-01025-7 (PMC9446771; doi:10.1186/s12912-022-01025-7)
Supplement: Supplementary file 2 — Additional file 2. The 28-items questionnaire at the end of item analysis step. [file 12912_2022_1025_MOESM2_ESM.docx]

Additional file 2: The 28-items questionnaire at the end of item analysis step

| No | Item | Strongly agree | Agree | Neither agree nor disagree | Disagree | Strongly disagree |
| --- | --- | --- | --- | --- | --- | --- |
| 1 | My physical health is endangered due to taking care of my patient. |  |  |  |  |  |
| 2 | Accompanying my patient in the hemodialysis center makes me tired. |  |  |  |  |  |
| 3 | Because of my patient, my diet has been disrupted. |  |  |  |  |  |
| 4 | Taking care of my patient has made me feel depressed. |  |  |  |  |  |
| 5 | I am constantly thinking about my patient. |  |  |  |  |  |
| 6 | I am worried that I have failed to take care of my patient. |  |  |  |  |  |
| 7 | I feel that I cannot do anything about my patient's problems. |  |  |  |  |  |
| 8 | I feel I can no longer continue to take care of my patient. |  |  |  |  |  |
| 9 | I do not sleep well because of taking care of my patient. |  |  |  |  |  |
| 10 | My patient's hemodialysis schedule determines my life plan. |  |  |  |  |  |
| 11 | I am strained that there is no one or center to answer my questions about my patient. |  |  |  |  |  |
| 12 | I am worried about the future of my life. |  |  |  |  |  |
| 13 | I am afraid of my patient’s future. |  |  |  |  |  |
| 14 | My recreation has decreased due to taking care of my patient. |  |  |  |  |  |
| 15 | My relationships with others have diminished because of caring for my patient. |  |  |  |  |  |
| 16 | I can travel less than before. |  |  |  |  |  |
| 17 | Taking care of my patient has negatively affected my job and business. |  |  |  |  |  |
| 18 | After starting hemodialysis, I am responsible for my patient and my own life. |  |  |  |  |  |
| 19 | I feel that others do not understand my problems in taking care of my patient. |  |  |  |  |  |
| 20 | The non-cooperation of other family members in caring has imposed a significant burden on me. |  |  |  |  |  |
| 21 | The weakness of the support system (government and charity) for the patient and caregiver has burdened me. |  |  |  |  |  |
| 22 | The hospital staff understands and empathizes with me about my patient. |  |  |  |  |  |
| 23 | Ward staff’s inadequate involvement has reduced my confidence in them. |  |  |  |  |  |
| 24 | The burden of caring for my patient has reduced my sexuality. |  |  |  |  |  |
| 25 | One of my difficulties is monitoring my patient’s medication and nutrition. |  |  |  |  |  |
| 26 | Being forced to accompany my patient (in a dialysis center, medical visits, laboratory, etc.) takes a lot of my time. |  |  |  |  |  |
| 27 | My patient's lack of full health insurance coverage has imposed a financial burden on me. |  |  |  |  |  |
| 28 | I cannot afford my patient’s care. |  |  |  |  |  |
